# Perceptions of harmfulness of heated tobacco and nicotine vaping products compared to cigarettes, and the association of advertising exposure on harm perceptions among adults who smoke in South Korea: Cross-sectional findings from the 2020 ITC Korea Survey

**DOI:** 10.18332/tid/170252

**Published:** 2023-09-28

**Authors:** Michelle R. Goulette, Shannon Gravely, Steve S. Xu, Gang Meng, Anne C.K. Quah, Sungkyu Lee, Sung-il Cho, Yeol Kim, Sujin Lim, Maansi Bansal-Travers, Andrew Hyland, Geoffrey T. Fong, Hong G. Seo

**Affiliations:** 1 Department of Health Behavior, Roswell Park Comprehensive Cancer Center, Buffalo, United States; 2 Department of Psychology, University of Waterloo, Waterloo, Canada; 3 Korea Center for Tobacco Control Research and Education, Seoul, Republic of Korea; 4 Graduate School of Public Health, Seoul National University, Seoul, Republic of Korea; 5 Graduate School of Cancer Science and Policy, National Cancer Center, Goyang-si, Republic of Korea; 6 National Tobacco Control Center, Korean Health Promotion Institute, Seoul, Republic of Korea; 7 School of Public Health Sciences, University of Waterloo, Waterloo, Canada; 8 Ontario Institute for Cancer Research, Toronto, Canada

**Keywords:** tobacco, cigarettes, heated tobacco products, nicotine vaping products, perceptions, harmfulness

## Abstract

**INTRODUCTION:**

Heated tobacco products (HTPs) and nicotine vaping products (NVPs) both are legal consumer products in the Republic of Korea. Little is known about perceptions of harmfulness of HTPs and NVPs relative to cigarettes in South Korea among adults who smoke, and how exposure to marketing may be associated with harmfulness perceptions.

**METHODS:**

This study used data from the 2020 International Tobacco Control (ITC) Korea Survey, and included 3713 adult (aged 19 years) cigarette smokers who were: 1) exclusive smokers (n=1845); 2) dual HTP + cigarette consumers (n=1130); 3) dual NVP + cigarette consumers (n=224); and 4) triple consumers (all three products, n=514). Weighted multinomial regression models were conducted to estimate smokers’ perceptions of harmfulness of HTPs and NVPs compared to cigarettes, NVPs to HTPs, and self-reported exposure to HTP/NVP advertising. Analyses compared the perceptions of harmfulness between the four different consumer groups, and tested whether exposure to HTP/NVP advertising was associated with perceptions of lower relative harm.

**RESULTS:**

Among all respondents, 27.5% believe that HTPs are less harmful than cigarettes and 23.4% believe that NVPs are less harmful than cigarettes. Exclusive cigarettes smokers were significantly less likely to perceive that HTPs and NVPs are less harmful than cigarettes compared to dual HTP + cigarette consumers, dual NVP + cigarette consumers, and triple consumers (all p<0.001). Half of respondents perceive NVPs as equally harmful as HTPs (14.1% perceive NVPs as more harmful than HTPs). Exposure to HTP/NVP advertising was associated with perceiving these products as less harmful than cigarettes.

**CONCLUSIONS:**

About one-quarter of Korean cigarette smokers perceive HTPs and NVPs as less harmful than cigarettes. Further investigation is required to understand how harm perceptions and HTP/NVP advertising are related to changes in product use, such as switching between products, using multiple products, or discontinuing all product use.

## INTRODUCTION

A new generation of non-combustible nicotine products has emerged in the last decade, such as nicotine vaping products (NVPs) or e-cigarettes. Scientific reviews have concluded that although NVPs are not harmless^1,2^, completely switching from cigarettes to NVPs can greatly reduce exposure to several toxicants, including carcinogens^3-5^. Less is known about the safety profile of the exclusive use of HTPs, but some evidence indicates that they may reduce consumers’ exposure to several chemicals found in cigarettes^6^.

HTPs and NVPs are legal in the Republic of Korea (hereinafter referred to as ‘South Korea’), and both products are used by a substantial percentage of the population, with greater prevalence among men and those who smoke cigarettes^7-10^. NVPs were first introduced in South Korea in 2007 and were initially advertised in South Korea as ‘incredible smoking cessation devices’, ‘a less harmful alternative to cigarettes’, and ‘healthy cigarettes’^11^. However, as marketing regulations became more restrictive^12^, NVP advertising became more focused on branding, design, and technology, and less focused on product risk^13,14^. In recent years, the South Korean government has discouraged NVP use^15^.

Following the successful national launch of IQOS by Philip Morris International (PMI) in Japan in 2016, IQOS was introduced to the South Korean market in May 2017. In 2018, Korea Tobacco & Ginseng Corporation and British American Tobacco introduced their own HTPs – ‘lil’ and ‘glo’, respectively. Similar to the marketing strategies seen in Japan, the tobacco industry marketed HTPs in South Korea as a ‘less harmful’ and as a ‘clean’ alternative to cigarettes. And while PMI and other tobacco companies have made claims that switching completely from cigarettes to a HTP would reduce health risk, the majority of South Korean HTP consumers also smoke cigarettes^16^.

Tobacco advertising, packaging, and health warnings communicate messages to the public about product characteristics^17^, in turn shaping consumers’ perceptions about tobacco/nicotine products, including health risks. In South Korea, HTP tobacco refills and NVP e-liquids containing nicotine extracted from tobacco leaves are subject to the same marketing regulations as tobacco cigarettes^18,19^. Advertisements of both products are allowed in retail stores and media channels, with some exceptions. Pictorial health warnings are also required, covering 50% of the front and back of packaging^20^. Tobacco packaging cannot include misleading descriptors such as ‘low tar’, ‘light’, or ‘mild’. Supplementary file Table 1 shows tobacco product regulations for tobacco advertising, promotion, and sponsorship (TAPS) and heath warning labels in South Korea.

Studies have shown that among people who smoke cigarettes and perceive NVPs as less harmful than cigarettes are more likely to use them^21,22^, including for smoking cessation purposes^23^. In Japan, cigarette smokers who reported having been exposed to marketing of HTPs, were more likely to be using them and perceive HTPs as less harmful^24^. There is, however, limited research on how adults who smoke in South Korea perceive the relative risks of HTP and NVPs to cigarettes, and whether marketing exposure might be associated with those perceptions. To our knowledge, only one study has examined tobacco users’ relative harm perceptions of HTPs and NVPs compared to cigarettes in South Korea, which found that about a quarter of adult tobacco product users perceived HTPs and NVPs to be less harmful than cigarettes (a majority believe that they are equally or more harmful than cigarettes)^25^. Thus, our study aimed to compare Korean adult cigarette smokers’ perceptions of relative harmfulness between HTPs, NVPs and cigarettes to the study by Kim et al.^26^. Additionally, we also examined perceptions of relative harmfulness of NVPs to HTPs, and tested whether exposure to HTP/NVP advertising via various marketing outlets was related to beliefs that they are less harmful than cigarettes compared to those who reported not being exposed to advertisements.

## METHODS

### Study design, setting, participants

Cross-sectional data for this study were from Wave 1 of the International Tobacco Control Korea (ITC KRA1) Survey (conducted in June 2020), a web-based study of 4794 adults (aged 19 years) recruited from Rakuten Insight’s web panel^26^. Those who exclusively smoke cigarettes, use HTPs and/or NVPs (at least weekly), former cigarette smokers, and non-nicotine product consumers were invited to participate and completed the online survey. The response rate was 15.2% and the cooperation rate was 97.4%. A detailed description of the sample and methods are reported in the Wave 1 Korea technical report^26^.

This cross-sectional study included 3713 adults who were smoking cigarettes at least weekly at the time of the survey, of whom 1845 were exclusive smokers, 1130 were dual HTP + cigarette consumers, 224 were dual NVP + cigarette consumers, and 514 were triple consumers (sample selection in Supplementary file Figure 1). The study protocol for involving human data was in accordance with the Declaration of Helsinki.

### Measures

All survey content was initially developed in English in collaboration between Korean and Canadian research team members. The final English survey was then translated into Korean by a professional translator at the survey firm (Rakuten Insight). The Korean translation was checked and verified by Korean researchers to meet the standards for the highest possible degree of clarity and accuracy and have the closest equivalence to the English survey content. The full survey can be found at: https://itcproject.org/surveys/republic-korea/kra1-cohort3/ .

*Independent variables*

All respondents were asked whether they were smoking cigarettes or using an NVP or HTP at the time of the survey. If respondents were smoking cigarettes at least weekly, then they were considered eligible for the study. The sample was divided into four groups based on their self-reported product use at the time of completing the survey:

1. Exclusive smokers: those who smoke cigarettes at least weekly (e.g. some days of the week or every day) and not using HTPs or NVPs;
2. Dual HTP + cigarette consumers: those who smoke cigarettes and use HTPs at least weekly (not using NVPs);
3. Dual NVP + cigarette consumers: those who smoke cigarettes and use NVPs at least weekly (not using HTPs); and
4. Triple consumers: those who use all three products at least weekly.

*Covariates*

Sex, age group, annual household income, and education level were used as covariates in this study.

*Outcome measures*

Supplementary file Table 2 describes the outcome measures with original survey response options. In brief, respondents were asked: 1) ‘Compared to smoking ordinary cigarettes, how harmful do you think it is to use a heated tobacco product?’; 2) ‘Compared to smoking ordinary cigarettes, how harmful do you think it is to use a liquid e-cigarette?’; and 3) ‘Compared to using a liquid e-cigarette, how harmful do you think using a heated tobacco product is?’. For questions 1 and 2, the outcome was dichotomized into: ‘they are less harmful than cigarettes’ versus ‘they are equally or more harmful than cigarettes/don’t know’. For question 3, the outcome was dichotomized into: ‘NVPs are less harmful than HTPs’ versus ‘NVPs are equally or more harmful than HTPs/‘don’t know’.

Respondents were also asked: ‘In the last 6 months have you noticed [heated tobacco products] [liquid e-cigarette products] being advertised in any of the following places?’: TV; radio; newspapers or magazines; posters or billboards; stores where tobacco is sold; stores where HTPs are sold; stores where NVPs are sold; social media; and bars or pubs. Respondents could select all that applied. Responses were dichotomized as: ‘Yes’ (if respondents reported noticing advertising for one or both products for each location) or ‘No’ (if respondents reported ‘no’ or ‘don’t know’ to both questions).

### Statistical analysis

Unweighted data were used to describe the study sample, overall, and by user group status. Chi-squared was used to test whether there were differences in sample characteristics between the four user groups. All subsequent analyses were weighted. A raking algorithm was used to calibrate the weights to target marginal joint population distributions of cigarette, HTP, and NVP use, geographical region, and demographic measures. All analyses were conducted using SAS Version 9.4. Statistical significance and confidence intervals were computed at the 95% confidence level, and all tests were two-tailed.

Three multinomial regression models were conducted to compute weighted and adjusted estimates for perceived relative harmfulness for HTPs relative to cigarettes, NVPs relative to cigarettes, and NVPs relative to HTPs. The outcomes included: ‘less harmful’ versus ‘equally/more harmful’ versus ‘don’t know’. The reference group used for this analysis was ‘equally/more harmful’. Each of the models compared the perceptions of harmfulness between the four different user groups and controlled for geographical region, education level, income, age, and sex.

Adjusted logistic regression analyses were conducted to test whether exposure (vs no exposure) to HTP/NVP advertising in each of the 10 locations was associated with perceptions of lower relative harmfulness of HTPs and NVPs compared to cigarettes, adjusting for region, age group, sex, education level, and income.

## RESULTS

Table 1 presents the (unweighted) characteristics of the study sample. In brief, the majority of the sample were men (79.8%), daily smokers (87.8%), aged 40–59 years (53.9%), had a higher level of education (80.1%) and a moderate household income (57.5%).

**Table 1:** Descriptive characteristics of the study sample of adults who smoke cigarettes (at least weekly), Republic of Korea, June 2020 (N=3713)

*Characteristics*	*Exclusive cigarette smokers (N=1845) %*	*HTP + cigarette consumers (N=1130) %*	*NVP + cigarette consumers (N=224) %*	Triple consumers* (N=514) %	*All respondents (N=3713) %*	*p*
**Sex**						<0.001
Male	84.0	78.0	76.8	70.2	79.8	
Female	16.0	22.0	23.2	29.8	20.2	
**Age** (years)						<0.001
19–29	9.7	8.9	20.1	15.2	10.8	
30–39	21.3	29.1	34.4	37.4	29.7	
40–59	57.6	54.7	43.8	43.0	53.9	
≥60	11.5	7.4	1.8	4.5	8.7	
**Education level**						<0.001
Low	1.0	0.4	0	0.4	0.7	
Moderate	23.6	12.2	22.8	13.8	18.7	
High	75.1	86.9	76.3	85.0	80.1	
Not reported	0.3	0.4	0.9	0.8	0.5	
**Income**						<0.001
Low	17.6	7.8	17.4	9.5	13.5	
Moderate	58.8	57.0	57.1	54.5	57.5	
High	21.3	34.1	22.8	35.2	27.2	
Not reported	2.4	1.2	2.7	0.8	1.8	
**Smoking frequency**						<0.001
Daily	90.9	87.2	81.7	80.5	87.8	
Weekly	9.1	12.8	18.3	19.5	12.2	
**HTP use frequency**						<0.001
Daily	0.0	66.1	0.0	64.2	36.2	
Weekly	0.0	33.9	0.0	35.8	31.6	
Not at all	100	0	100.0	0.0	32.2	
**NVP use frequency**						<0.001
Daily	0	0	49.1	47.1	15.8	
Weekly	0	0	50.9	52.9	30.3	
Not at all	100	100	0	0	54.0	

HTP: heated tobacco product. NVP: nicotine vaping product.

* Triple consumers: those reporting using all three products at least weekly. Data are unweighted and unadjusted. Chi-squared tests were utilized to derive p values. Annual household income (Korean Won): low, <10 million (US$7700); moderate, 10–75 million (US$58000); high, ≥75 million; and ‘not reported’. Education: low, <high school; moderate, high school and some college; high, ≥undergraduate degree; and ‘not reported’.

### Perceptions of relative harmfulness

Table 2 presents estimates for relative harmfulness perceptions among all respondents. The majority of respondents reported that they perceive HTPs and NVPs as equally as harmful as cigarettes (53.7% and 49.9%, respectively), and NVPs as equally harmful as HTPs (55.4%).

**Table 2:** Perceived harmfulness of heated tobacco products relative to cigarettes, nicotine vaping products relative to cigarettes, and nicotine vaping products relative to heated tobacco products among adults who smoke cigarettes, Republic of Korea, June 2020 (N=3173)

	Less harmful % (95% CI)	Equally harmful % (95% CI)	More harmful % (95% CI)	Don’t know % (95% CI)
HTPs relative to cigarettes	27.6 (26.1–28.9)	53.7 (52.1–55.3)	7.6 (06.7–08.4)	11.2 (10.1–12.2)
NVPs relative to cigarettes	23.4 (22.0–24.7)	49.9 (48.4–51.6)	12.8 (11.7–13.8)	13.9 (12.8–14.9)
NVPs relative to HTPs	14.1 (12.9–15.2)	55.4 (53.8–57.0)	14.9 (13.8–16.0)	15.6 (14.4–16.7)

Multinomial regression models were conducted to compute weighted and adjusted estimates for perceived relative harmfulness for HTPs relative to cigarettes, NVPs relative to cigarettes, and NVPs relative to HTPs. The models adjusted for geographical region, income, education level, age, and sex. HTPs: heated tobacco products. NVPs: nicotine vaping products.

**Figure 1:**
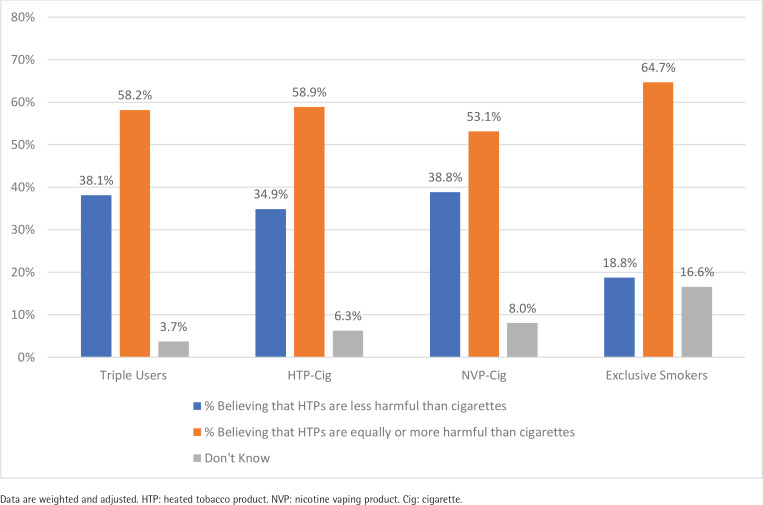
Perceived harmfulness of HTPs compared to cigarettes among adults who smoke cigarettes, Republic of Korea, June 2020 (N=3137)

Figure 1 presents the estimates across user groups for perceived harmfulness of HTPs compared to cigarettes. The majority (61.3%) of all respondents perceive HTPs to be equally or more harmful compared to cigarettes. Exclusive smokers were significantly less likely to perceive that HTPs are less harmful than cigarettes (18.9%) compared to dual HTP + cigarette consumers (34.9%, p<0.001), dual NVP + cigarette consumers (38.8%, p<0.001) and triple consumers (38.1%, p<0.001). Perceptions of lower harmfulness did not differ between triple consumers and dual NVP + cigarette consumers (p<0.75).

**Figure 2:**
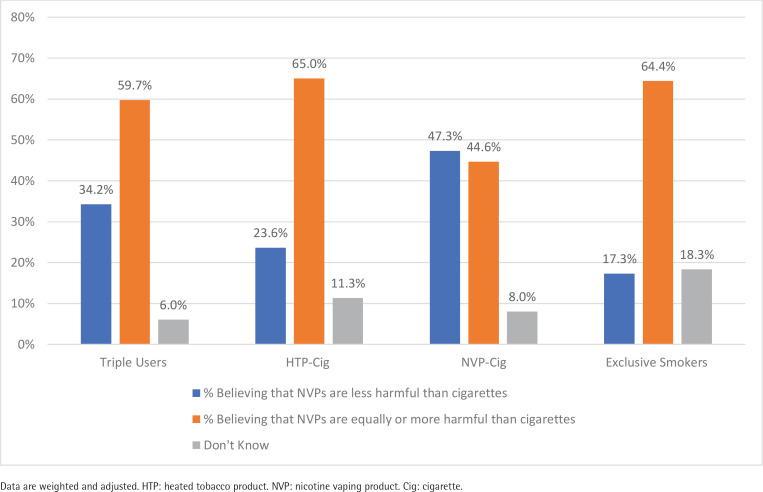
Perceived harmfulness of NVPs compared to cigarettes among adults who smoke cigarettes, R epublic of Korea, June 2020 (N=3137)

Figure 2 presents the estimates across user groups for perceived harmfulness of NVPs compared to cigarettes. The majority of all respondents (62.7%) reported that they perceive NVPs as equally or more harmful compared to cigarettes. Exclusive smokers were less likely to believe that NVPs are less harmful than cigarettes (17.3%) compared to dual NVP + cigarette consumers (47.3%, p<0.001), dual HTP + cigarette consumers (23.6%, p<0.01) and triple consumers (34.2%, p<0.001). Triple consumers were less likely to report that NVPs are less harmful than cigarettes compared to dual NVP + cigarette consumers (p<0.01), but more likely than dual HTP + cigarette consumers (p<0.001).

**Figure 3:**
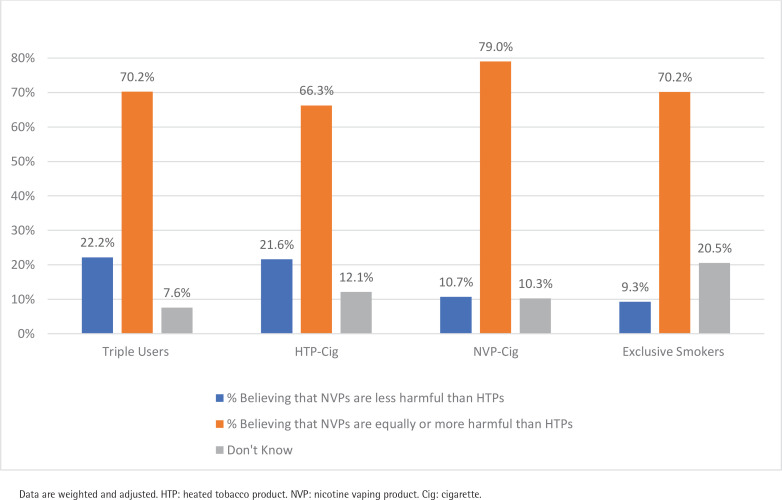
Perceived harmfulness of NVPs compared to HTPs among adults who smoke cigarettes, Republic of Korea, June 2020 (N=3137)

Figure 3 presents the estimates across user groups for perceived harmfulness of NVPs compared to HTPs. Among all respondents, 69.5% perceive that NVPs are equally or more harmful than HTPs. Exclusive smokers were less likely to believe that NVPs are less harmful than HTPs (9.3%) compared to dual HTP + cigarette consumers (21.6%, p<0.001), and triple consumers (22.1%) (all p<0.001), but did not differ from dual NVP + cigarette consumers (10.7%, p=0.16). Triple consumers were more likely to report that NVPs are less harmful than HTPs compared to dual NVP + cigarette consumers (p<0.01), and dual HTP + cigarette consumers (p<0.01).

The associations of different marketing exposures and perceptions of relative harm of HTPs and NVPs compared to cigarettes are presented in Table 3. In summary, exposure (vs no exposure) to marketing across all advertising platforms, with the exception of television, were significantly associated with perceiving HTPs and NVPs as less harmful than cigarettes (all p<0.001). The three locations of exposure that were associated with the highest odds of perceiving that HTPs/NVPs are less harmful than cigarettes were: transportation adds (unexposed: 26.0% vs exposed: 46.1%; AOR=2.44; 95% CI: 1.71–3.48), newspapers/magazines (unexposed: 25.0% vs exposed: 40.2%; AOR=2.01; 95% CI: 1.50–2.69) and stores where tobacco is sold (unexposed: 13.1% vs exposed: 35.2%; AOR=1.90; 95% CI: 1.46–2.45).

**Table 3:** Associations between exposure to heated tobacco/nicotine vaping product advertising locations in the past 6 months and the perception that heated tobacco and nicotine vaping products are less harmful than cigarettes among adults who smoke, Republic of Korea, June 2020 (N=3713)

*Advertising location (Exposure = yes)*	*HTP/NVPs are less harmful than cigarettes %*	*AOR*	*95% CI*	*p*
**Television**				0.22
Yes, exposed (N=999)	31.3	1.20	0.90–1.61	
No, not exposed (N=2714) (Ref.)	27.5	1		
**Radio**				0.02
Yes, exposed (N=547)	37.0	1.56	1.08–2.25	
No, not exposed (N=3166) (Ref.)	27.4	1		
**Newspapers or magazines**				<0.001
Yes, exposed (N=984)	40.2	2.01	1.50–2.69	
No, not exposed (N=2729) (Ref.)	25.0	1		
**Posters or billboards**				<0.001
Yes, exposed (N=1378)	35.6	1.68	1.28–2.18	
No, not exposed (N=2335) (Ref.)	24.8	1		
**Stores where tobacco is sold**				<0.001
Yes, exposed (N=1912)	35.2	1.90	1.46–2.45	
No, not exposed (N=1801) (Ref.)	22.3	1		
**Stores where HTPs are sold**				<0.001
Yes, exposed (N=1660)	34.4	1.67	1.29–2.16	
No, not exposed (N=2053) (Ref.)	23.9	1		
**Stores where NVPs are sold**				<0.001
Yes, exposed (N=1203)	35.8	1.68	1.30–2.20	
No, not exposed (N=2510) (Ref.)	25.0	1		
**Social media**				<0.001
Yes, exposed (N=1497)	35.4	1.69	1.31–2.20	
No, not exposed (N=2216) (Ref.)	24.4	1		
**Bars or pubs**				<0.001
Yes, exposed (N=1404)	34.9	1.60	1.23–2.09	
No, not exposed (N=2309) (Ref.)	25.1	1		
**Transportation**				<0.001
Yes, exposed (N=614)	46.1	2.44	1.71–3.48	
No, not exposed (N=3099) (Ref.)	26.0	1		

AOR: adjusted odds ratio. Adjusted logistic regression analyses were used to test whether exposure (vs no exposure) to heated tobacco product/nicotine vaping product advertising in each of the 10 locations was associated with perceptions of lower relative harmfulness of heated tobacco and nicotine vaping products compared to cigarettes. Data are weighted and adjusted for geographical region, education level, user group, age, and sex. HTPs: heated tobacco products. NVPs: nicotine vaping products. The p-values were derived from multivariable logistic regression models.

## DISCUSSION

This study examined South Korean adult smokers’ perceptions of the harmfulness of HTPs and NVPs compared to cigarettes, and to each other, and tested whether exposure to various advertising mediums was associated with believing that HTPs/NVPs are less harmful than cigarettes. We found that about a quarter of smokers perceived HTPs and NVPs to be less harmful than cigarettes, which is consistent with the study by Kim et al.^25^ who found in 2019 that 26% of adult tobacco users believed that HTPs and NVPs were less harmful than cigarettes. Also consistent with Kim et al.^25^ and smokers in Japan^24^, we found that exclusive smokers (non-HTP and non-NVP consumers) were less likely to hold this believe compared to those who use the products. However, in contrast to our findings, Kim et al.^24^ found a much higher proportion of tobacco users who believe that HTPs (38%) and NVPs (41%) are more harmful than cigarettes, whereas we found that the majority (55%) believed that HTPs and NVPs are equally as harmful (fewer than 10% believed that HTPs were more harmful than cigarettes and 13% believed NVPs were more harmful than cigarettes in our study). A similar proportion of respondents in each of the studies reported they did not know. Thus, although there is evidence that completely switching from cigarettes to HTPs or NVPs likely reduces exposure to several cigarette-related toxicants, the majority of Korean smokers in both our study and in Kim et al.^25^ believe that HTPs/NVPs are not less harmful than cigarettes, regardless of whether they use them or not. As it has been shown that smokers’ perceptions of the harmfulness of NVPs relative to cigarettes predicted the respective product use when trying to quit cigarette smoking^23^, many Korean smokers may be reluctant to use HTPs or NVP to quit smoking, or as a complete substitute for cigarettes, although this has not yet been tested in South Korea.

Cytotoxicity studies have found that harmful chemicals are lower in HTPs than cigarettes, but higher in HTPs relative to NVPs^4,27^. Thus, in addition to examining relative risk perceptions between cigarettes and HTPs/NVPs, we also examined whether smokers perceive that HTPs and NVPs differ in harmfulness, which was not addressed in the Kim et al.^25^ study. We found that about half (55%) of Korean smokers perceive HTPs and NVPs as being equally harmful (70% reported believing that NVPs are equally/more harmful than HTPs), with only 14% of all respondents correctly perceiving that HTPs are more harmful than NVPs. Another ITC study that assessed relative risk between HTPs and NVPs among Canadian nicotine users found that a similar proportion of respondents reported believing that HTPs are more harmful than NVPs (17%)^28^. However, a higher proportion of Canadian respondents reported that HTPs are less harmful than NVPs (23%) in the Canada study compared to Korean respondents (15%). The results from these two studies appear to show perceptions of harmfulness are not consistent with the current evidence that NVPs expose consumers to lower levels of toxicants than HTPs.

HTPs have been launched and marketed in several countries; however, HTP market growth has generally been slow in most countries, with the exception of Japan and Korea^29^. Comparing perceptions of relative risk between HTPs, NVPs, and cigarettes across studies with similar study measures and sampling frames in countries with similar or differing levels of success and policy regulations, including and marketing restrictions is warranted. For example, analyses of our ITC Japan Survey found that in 2018, 48% of smokers perceived HTPs to be less harmful than cigarettes^24^; however, this declined over time to 28.3% in 2020^30^. We have speculated that this large reduction in perceptions of relative harmfulness among Japanese smokers may have been due to changes in Japan’s HTP policies: in 2018, there were no restrictions on use or marketing of HTPs^31^, but by 2020, HTPs were banned in key public places and text health warnings were mandated on 1 April 2020 on HTP tobacco heat stick packaging^32^. Thus, after Japan introduced stricter regulations, perceptions were similar between Japan and South Korea in 2020.

When we compared our estimates to other ITC countries, we found stark differences between this study and the Santos et al.^28^ study in Canada (which regulates HTPs and NVPs under the Tobacco and Vaping Products Act^33^). We found that a much greater percentage of Canadian respondents reported believing that HTPs (48%) and NVPs (66%) are less harmful than cigarettes. Gravely et al.^22^ assessed relative risk perceptions among smokers from six European countries (where NVPs are regulated under the EU Tobacco Products Directive^34^) and found that a quarter of smokers perceived NVPs as less harmful than cigarettes, with some variations across the countries (ranging from 22% in Spain to 34% in Hungary). The differences between Canada and South Korea may be that the Canadian government appears to have taken a different approach to NVPs, in that Health Canada’s Tobacco Strategy stated that NVPs could be helpful for smokers attempting to quit, particularly if they were unsuccessful with other medically approved cessation aids^35,^ but that youth and never smokers should not use them, whereas the EU has taken a less supportive stance^36^, which is more aligned with the position of the South Korean government.

Finally, the results from our study demonstrate that smokers who were exposed to HTP/NVP advertising were more likely to hold the belief that these products are less harmful than cigarettes. We also found this in the ITC Japan Survey^24^. However, both of these studies were cross-sectional so we cannot determine if risk perceptions preceded or followed exposure. We also found in the Japan study that HTP consumers were more likely to be exposed to advertising than non-HTP consumers, likely because they visit locations where HTPs are sold, and/or they are more aware of the product. However, it is notable that even after exposure to advertising in South Korea, the minority of respondents perceived HTP/NVPs as less harmful than cigarettes, which may be due to the industry’s shift in focus from product risk to marketing product branding, design, and technology. As to whether marketing strategies change beliefs about the relative risk of HTPs and NVPs requires further study using longitudinal cohort designs.

### Limitations

While this study has many strengths, including the large sample size of representative cigarette smokers and HTP and NVP consumers in South Korea, there are limitations to consider. First, this study is cross-sectional; thus, we do not attempt to imply causality. Second, the assessment of exposure to advertising outlets could be subject to recall bias. Lastly, the generalizability of findings is limited to only those who use HTPs and/or frequently (at least weekly) and/or who smoke cigarettes, and are not representative of the general South Korean population or those who may have experimented with HTPs and/or NVPs.

## CONCLUSIONS

Even though current existing scientific data show lower exposure to toxic substances from HTPs and NVPs than from cigarettes, only a quarter of South Korean smokers believe that HTPs and NVPs are less harmful than cigarettes. Those who used HTPs and/or NVPs were more likely than non-consumers to hold the belief that they are less harmful. Most respondents perceive that HTPs and NVPs have a similar risk profile when compared to each other, with few believing that NVPs are less harmful than HTPs. Future research should identify both the absolute and relative exposure to toxins and health risks from cigarettes, HTPs, and NVPs. Risk communication about HTPs and NVPs should align with the best-available scientific evidence.

## Data Availability

The data that support the findings of this study are available from the ITC Project at the University of Waterloo but restrictions apply to the availability of these data, which were used under license for the current study, and so are not publicly available. Data are, however, available from S. Gravely upon reasonable request and with permission from the ITC Project and the lead researcher(s) in the country where the data are from. The criteria for data usage approval and the contents of the Data Usage Agreement are described online (http://www.itcproject.org).
