# Supplementary material for: Perceptions of harmfulness of heated tobacco and nicotine vaping products compared to cigarettes, and the association of advertising exposure on harm perceptions among adults who smoke in South Korea: Cross-sectional findings from the 2020 ITC Korea Survey
Source: Tob Induc Dis. 2023 Sep 28;21:121. doi: 10.18332/tid/170252 (PMC10535845; doi:10.18332/tid/170252)
Supplement: Supplementary file 1 [file TID-21-121-s1.pdf]

Supplemental Table 1. Tobacco product regulations for tobacco advertising, promotion, and sponsorship (TAPS) and heath warning labels

	TAPS bans (FCTC Article 13): Location covered by a ban							FCTC Article 11
	**TV and Radio**	**Print Ads: Magazine and Newspapers**[†]	**Billboard/ Outdoor Advertising**	**POS**	**Internet**	**Free distribution in mail or through other means**	**Promotional Discounts**	**Health Warning Labels**
**Cigarettes**	Yes	No	No	Yes	No	No	No	Picture/graphic warnings cover 50% of the front and back (10 different warnings) 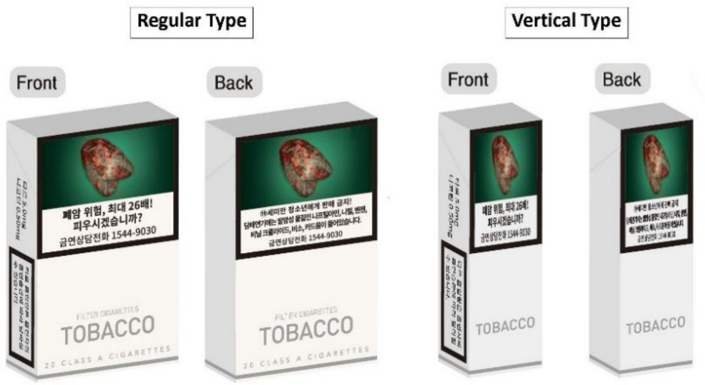

	TAPS bans (FCTC Article 13): Location covered by a ban							FCTC Article 11
	**TV and Radio**	**Print Ads: Magazine and Newspapers**[†]	**Billboard/ Outdoor Advertising**	**POS**	**Internet**	**Free distribution in mail or through other means**	**Promotional Discounts**	**Health Warning Labels**
**HTPs (tobacco-containing heat sticks)***	Yes	No	No	Yes	No	No	No	Picture/graphic warnings cover 50% of the front and back (1 warning) 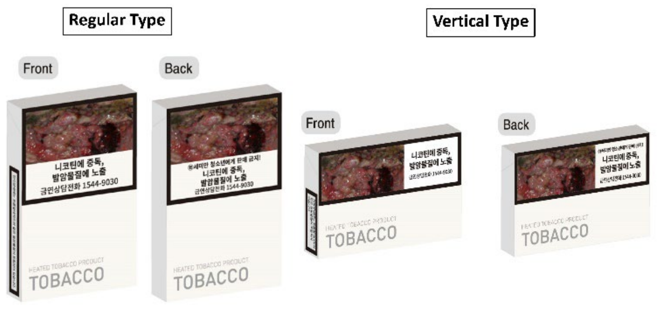

<table>
<tr><th></th><th colspan="7">TAPS bans (FCTC Article 13): Location covered by a ban</th><th>FCTC Article 11</th></tr>
<tr><th></th><th>TV and Radio</th><th>Print Ads: Magazine and Newspapers[†]</th><th>Billboard/ Outdoor Advertising</th><th>POS</th><th>Internet</th><th>Free distribution in mail or through other means</th><th>Promotional Discounts</th><th>Health Warning Labels</th></tr>
<tr><td>NVPs (nicotine e-liquid packages)*</td><td>Yes</td><td>No</td><td>No</td><td>Yes</td><td>No</td><td>No</td><td>No</td><td>Picture/graphic warnings cover 50% of the front and back (2 warnings)

The e-liquid bottle contains the picture warning on the front of the bottle.

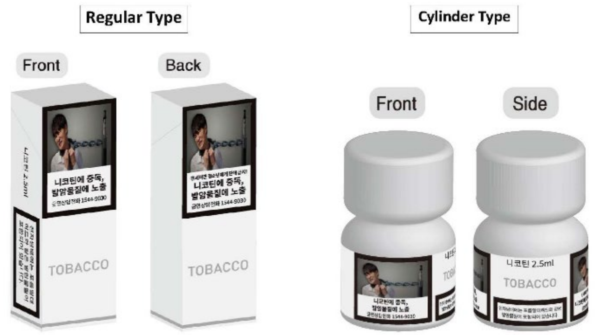
</td></tr>
</table>

*Devices are excluded from tobacco product regulations; † If magazines and newspapers are particularly targeting women and youth, all TAPS for cigarettes, HTP and NVP are banned. NVPs: If liquid nicotine is extracted from tobacco roots and stems, and synthetic nicotine is used, they are not regulated as tobacco products. POS: Point of sale (retail) advertising. Health warning label images differ between cigarettes, HTPs and NVPs. Health warning information and images for tobacco packaging are from: The 2nd Manual for Marking the Warning Picture and Phrases on Packaging of Tobacco, Ministry of Health and Welfare & the National Tobacco Center.

Supplemental Table 2. Outcome measures used in the current study: Wave 1 of the International Tobacco Control Korea (ITC KRA1) Survey (conducted in June 2020).

Outcome variables

1. Perceived Harmfulness of HTPs/NVPs compared to cigarettes was assessed using the following questions:

(a) *"Compared to smoking ordinary cigarettes, how harmful do you think it is to use a heated tobacco product?"*
(b) *"Compared to smoking ordinary cigarettes, how harmful do you think it is to use a liquid e-cigarette?"* Response options for 1a and 1b were :

- Much less harmful than smoking ordinary cigarettes
- Somewhat less harmful than smoking ordinary cigarettes
- Equally harmful to smoking ordinary cigarettes
- Somewhat more harmful than smoking ordinary cigarettes
- Much more harmful than smoking ordinary cigarettes
- Declined
- Don't know

2. Perceived Harmfulness of HTPs to NVPs was assessed using the following question: *"Compared to using a liquid e-cigarette, how harmful do you think using a heated tobacco product is?"* Response options included:

- Much less harmful than using liquid e-cigarettes
- Somewhat less harmful than using liquid e-cigarettes
- Equally harmful as using liquid e-cigarettes
- Somewhat more harmful than using liquid e-cigarettes
- Much more harmful than using liquid e-cigarettes
- Declined
- Don't know

3. Advertising exposure to HTPs and NVPs was assessed using the following two questions: *"In the last 6 months have you noticed heated tobacco products being advertised in any of the following places?"* and "*In the last 6 months have you noticed liquid e-cigarette products being advertised in any of the following places?" TV; Radio; Newspapers or Magazines; Posters or Billboards; Stores where tobacco is sold; Stores where HTPs are sold; Stores where NVPs are sold; Social Media; Bars or Pubs; Transportation.* Response options included:

- Yes
- No
- Declined
- Don't know

© **2023 Goulette M.R. et al.**
